# Prenatal detection of distal 1q21.1q21.2 microduplication with abnormal ultrasound findings: Two cases report and literature review

**DOI:** 10.1097/MD.0000000000024227

**Published:** 2021-01-08

**Authors:** Hongguo Zhang, Fagui Yue, Xinyue Zhang, Jing He, Yuting Jiang, Ruizhi Liu, Yang Yu

**Affiliations:** a Center for Reproductive Medicine, Center for Prenatal Diagnosis, First Hospital; b Jilin Engineering Research Center for Reproductive Medicine and Genetics, Jilin University, Changchun, China.

**Keywords:** 1q21.1 duplication, chromosomal microarray analysis, phenotypic diversity, ultrasound findings

## Abstract

**Rationale:**

1q21.1 duplication is an uncommon chromosomal submicroscopic imbalance which is associated with growth/mental retardation, dysmorphic features, autism, multiple congenital and neuropsychiatric disorders.

**Patient concerns:**

Two pregnant women underwent amniocentesis for cytogenetic analysis and chromosomal microarray analysis (CMA) following abnormal ultrasound findings. Case 1 presented short nasal bone and case 2 showed absent nasal bone, ventricular septal defect and umbilical cord circling in ultrasonic examination.

**Diagnoses:**

G-banding analysis showed that the two fetuses presented normal karyotypic results while CMA detected 1.796 Mb (case 1) and 1.242 Mb (case 2) microduplications in the region of 1q21.1q21.2 separately. Furthermore, the CMA also revealed a 1.2 Mb microdeletion of 8p23.3 in case 1.

**Interventions:**

The couple in case 1 chose to terminate the pregnancy, while the couple in case 2 continued the pregnancy and finally delivered a male infant who presented low nasal bridge and ventricular septal defect.

**Outcomes:**

The 1q21.1q21.2 duplications in our report were located in the distal 1q21.1 region, overlapping with 1q21.1 duplication syndrome. Case 2 was the first reported live birth with 1q21.1 duplication according to prenatal CMA detection in China.

**Lessons:**

The genotype-phenotype of 1q21.1 duplication is complicated due to the phenotypic diversity, incomplete penetrance, and lack of obvious characteristics. So it is difficult to predict the postnatal development and health conditions clinically. Hence, long term follow up is necessary for newborn infants with 1q21.1 duplication, irrespective of whether the duplication is *de novo* or inherited.

## 1 Introduction

Chromosomal microdeletions and microduplications, making up a fraction of copy-number variants (CNVs), have long been associated with mental retardation, congenital abnormalities, autistic disorders and other genetic disorders. As molecular techniques with high resolution, chromosomal microarray analysis (CMA) play a critical role in detecting these chromosomal submicroscopic imbalances, which are too small to be identified through conventional karyotype analysis.^[1]^ The deleted/duplicated size, locus and dosage-sensitive genes involved have close correlation with the abnormal manifestations in clinic.^[2,3]^ Common CNVs associated with fetal anomalies include 22q11.21, 17q12, 16p13.11, 1q21.1, 15q13.3, and 10q21.1.^[4]^

Chromosomal 1q21.1 microdeletions/duplications are rare aberrations of chromosome 1. Patients with CNVs in 1q21.1 locus share similar phenotypes with 22q11.2 deletion syndrome, and they usually exhibit variable features, characterized by congenital abnormalities, intellectual disability, dysmorphic features, schizophrenia, autism and normal phenotypes.^[2,5–7]^ Currently, it is difficult to assess the pathogenicity and clinic significance for cases carrying 1q21.1 microdeletions/duplications.

For prenatal samples presenting karyotypic results, sub-chromosomal gains and losses of clinic significance can be detected in approximately 1% of structurally normal pregnancies and 6% with structural malformations.^[1]^ Herein, we present two cases of prenatal diagnostic 1q21.1 microduplication exhibiting abnormal ultrasound findings. We also present a review on the clinical data involving similar 1q21.1 microduplication, and delineated the genotype-phenotype correlations.

## 2 Methods

Our study protocol was approved by the Ethics Committee of the First Hospital of Jilin University (No.2019–281), and the written informed consents were obtained from the couples for publication of this case report and accompanying images.

### 2.1 Cytogenetic analysis

Chromosomal karyotypic analysis with a resolution of 300 to 400 bands was performed on G-band metaphases prepared from cultured aminotic fluid cells and peripheral blood cells according to standard protocols in our laboratory. Twenty metaphases were analyzed for all samples. The International System for Human Cytogenetic Nomenclature nomenclature was used to describe all karyotypes.^[8]^

### 2.2 CMA

Genomic DNA was extracted from 10 mL uncultured amino fluid cells using the QIAamp DNA Mini Kit (QIAGEN, Hilden, Germany) according to the manufacturer's protocol. The CMA was performed using the CytoScan 750K array (Affymetrix, Santa Clara, CA, USA), which included genomic DNA extraction, digestion and ligation, PCR amplification, PCR product purification, quantification and fragmentation, labeling, array hybridization, washing and scanning. Thresholds for genome-wide screening were set at ≥200 kb for gains, ≥100 kb for losses. The detected copy number variations were comprehensively estimated by comparing them with published literature and the public databases: (1) Database of Genomic Variants (http://dgv.tcag.ca/dgv/app/home), (2) DECIPHR (http://decipher.sanger.ac.uk/), (3) ISCA https://www.iscaconsortium.org/), (4) ECARUCA (http://www.ecaruca.net) and (5) online Mendelian inheritance in man (OMIM) (http://www.ncbi.nlm.nih.gov/omim).

## 3 Case presentation

### 3.1 Case 1

A 23-year-old, gravida 1, para 0, pregnant woman underwent ultrasound examination at 25 weeks of gestation, which indicated a short nasal bone in the fetus. Subsequently, the woman underwent amniocentesis for cytogenetic analysis and CMA detection. G-banding analysis showed that the karyotype of the fetus was 46, XY, but CMA revealed a 1.796Mb duplication in the region of 1q21.1q21.2 and a 1.2 Mb microdeletion in the region of 8p23.3. Meanwhile, the couple also accepted karyotypic analysis. The husband's karyotype was 46, XY, t(1;5)(q25;q33) while the wife's was 47, XXX. Finally, the couple chose to terminate the pregnancy according to genetic counseling based upon abnormal CMA results.

### 3.2 Case 2

A 29-year-old, gravida 2, para 0, abortus 1, pregnant woman underwent ultrasound examination at 23 weeks of gestation, which showed absent nasal bone, ventricular septal defect and umbilical cord circling in the fetus. Afterwards, the woman underwent amniocentesis for cytogenetic analysis and CMA detection. The karyotype of the fetus was identified as 46, XY. However, CMA detected a 1.242 Mb duplication in the region of 1q21.1 q21.2. In order to identify whether the 1q21.1 microduplication of the fetus was *de novo* or parentally inherited, the couple accepted CMA after informed consent. It turned out that case 2 inherited the 1q12.1 microduplication from the father with normal phenotype. Based upon genetic counseling, the couple continued the pregnancy and delivered a male infant at 41 weeks gestation, whose birth weight was 3,700 g and length was 51 cm. We followed- up on the postnatal health conditions. The infant presented low nasal bridge and ventricular septal defect, and no other apparent abnormalities were observed till now.

In our study, the couples were nonconsanguineous and healthy. There was no family history of diabetes mellitus or congenital malformations in the couples. These couples denied any exposure to alcohol, teratogenic agents, irradiation, or infectious diseases during this pregnancy.

## 4 Discussion

In our study, we report two prenatal cases with 1q21.1 duplication accompanied by abnormal ultrasound findings. Case 1 presented short nasal bone, with a 1.796 Mb microduplication of 1q21.1q21.2 and a 1.2 Mb microdeletion in 8p23.3. Case 2 showed absent nasal bone and ventricular septal defect, with a 1.242 Mb microduplication of 1q21.1q21.2. The duplicated regions in both cases overlapped with 1q21.1 duplication syndrome. To our knowledge, case 2 was the first live birth with 1q21.1 duplication according to prenatal CMA detection in China.

Chromosomal duplications can lead to different genetic disorders.^[4]^ 1q21.1 duplication syndrome (OMIM: 612475), an uncommon chromosomal submicroscopic imbalance, is associated with variable features characterized by growth/mental retardation, dysmorphic features, macrocephaly, multiple congenital malformations, autism, and neuropsychiatric anomalies.^[6,7,9]^ Currently, the incidence of this chromosomal duplication is not described. However, 1q21.1 microduplication was observed in about 0.03% of adults.^[10]^ The frequencies in patients presenting mental retardation, autism and/or congenital anomalies was between 0.103% and 0.172%.^[6,7]^ Multiple low copy repeats located in the chromosome 1q21.1 could cause this region susceptible to non-allelic homologous recombination, which would lead to genomic deletions and duplications.^[11]^ Two distinctive regions are included in the chromosomal 1q21.1 region: the proximal region which extends from breakpoints (BPs) 2 to BP3 spans about 0.2 Mb (chr1: 145.4–145.6 Mb, GRCh37/hg19) and distal region which extends from BP3 to BP4 spans about 1.35 Mb (chr1: 146.5–147.9 Mb, GRCh37/h19).^[12,13]^

**Figure 1:**
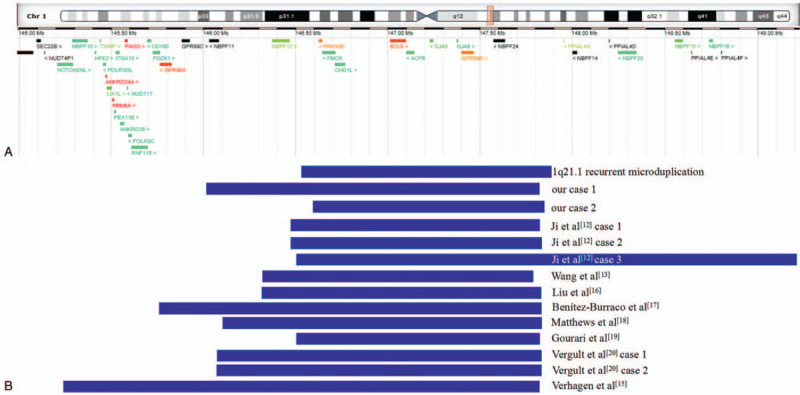
Scale representation of the duplicated region in the long arm of chromosome 1q21.1(https://decipher.sanger.ac.uk/): (A) Genes involved in the 1q21.1 locus; (B) Duplicated fragments in our cases and similar duplications in previous literature.

The duplicated regions in our study are located in the distal 1q21.1 region, spanning from BP3 to BP4. To better characterize the genotype–karyotype correlations of distal 1q21.1 microduplication, we summarized the clinical manifestations of prenatal/postnatal cases involving similar 1q21.1 duplication with our cases according to literature review (Table 1).^[12–20]^ Meanwhile, we also made a direct comparison focusing on these cases encompassing similar 1q21.1q21.2 duplication (Fig. 1). Most duplications were located in the region of 1q21.1q21.2(11/14), with the remainder in 1q21.1 (3/14). All 1q21.1 microduplications were varied in size, from 258 kb to 2.69 Mb. The age of these cases was ranging from fetus to 12 years: 4/14 cases were *de novo*, 7/14 cases were parentally inherited, and 3/14 cases were not available. 7/14 (No. 1–5, 13 and 14) were prenatal cases, and 7/14 (No. 6–12) were postnatal cases. All seven prenatal cases exhibited diverse ultrasound abnormalities: abnormal nasal bone was observed in 4/7 cases (No.1, 3, 13 and 14) and ventricular septal defect were shown in 3/7 cases (No. 4, 5 and 14). 5/7 couples finally chose termination of pregnancy. Since reports on prenatal phenotypes of 1q21.1 microduplications were limited, comprehensive genetic counseling of 1q21.1 duplication in prenatal cases should be taken seriously to avoid unnecessary abortions. For the postnatal cases, they presented a variety of clinical features with low specificity: mental/motor retardation, congenital anomalies, autism, finger/skeletal anomalies, poor language skills, dysmorphic features and psychiatric disorders, overlapping with part clinical manifestations of 1q21.1 duplication syndrome, which demonstrated the phenotypic diversity for 1q21.1 duplication. Overall, irrespective of whether the 1q21.1 duplications are parentally inherited from seemingly normal couples or *de novo*, regular follow up on their growth and health condition is necessary.

**Table 1:** Clinical manifestations of cases with similar 1q21.1 microduplication to our cases.

No.	Sex/age	Duplicated region	Duplicated size	Inheritance	Karyotype	Chromosome microarray results (hg19)	Referred critical gene	Clinical manifestation	References
1	M/TOP	1q21.1q21.2	1.34Mb	de novo	46, XX	1q21.1q21.2 (146,476,526–147,820,342) × 3	GJA5; GJA8	G2P1; absent nasal bone	Ji et al ^[12]^ case 1
2	F/TOP	1q21.1q21.2	1.35Mb	Maternally inherited	46, XY	1q21.1q21.2 (146,476,526–147,826,789) × 3	GJA5; GJA8	G1P0; duodenal atresia	Ji et al ^[12]^ case 2
3	F/TOP	1q21.1q21.2	2.69Mb	de novo	46, XY	1q21.1q21.2 (146,510,112–149,205,098) × 3	GJA5; GJA8	G2P0; absent nasal bone	Ji et al ^[12]^ case 3
4	F/n.r.	1q21.1	258 kb	de novo	46, XY	1q21.1 (144,337,316–144,595,988) × 3	PPIAL4B	G2P0; bilateral polycystic kidney; oligohydramnios; VSD	Liao et al ^[14]^
5	M/TOP	1q21.1	2.6 Mb	Maternally inherited	46, XY	1q21.1 (145,243,316–147,814,694) × 3	HFE2; PEX11B; RBM8A; GJA5; GJA8	fetal overgrowth; congenital heart malformation: dilated ventricles; VSD; dilated main pulmonary artery and aorta	Verhagen et al ^[15]^
6	F/9m6d	1q21.1	1.46 Mb	de novo	46, XX	1q21.1 (146,324,068–147,786,706) × 3	GJA5; GJA8	TOF combined with acleistocardia	Wang et al ^[13]^
7	F/6m9d	1q21.1q21.2	1.46 Mb	n.a.	46, XX	1q21.1q21.2 (146,324,068–147,821,717) × 3	GJA5; GJA8	TOF and patent foramen ovale	Liu et al ^[16]^
8	M/5y6m	1q21.1q21.2	2.1 Mb	Maternally inherited	46, XY	1q21.1q21.2 (145,764,455–147,824,207) × 3	GJA5; GJA8	non-symptomatic mother; cognitive delay and behavioral disturbances	Benítez-Burraco et al ^[17]^
9	M/12	1q21.1q21.2	1.73 Mb	Paternally inherited	n.a.	1q21.1q21.2 (146,105,170–147,823,369) × 3	GJA5; GJA8	Developmental delay; calf atrophy; dolichocephaly; hypertelorism; macrostomia; disordered eye movement; significant drooling; cerebral palsy; motor delay; speech delay; spastic paraplegia; autism	Matthews et al ^[18]^
10	F/10	1q21.1q21.2	1.3 Mb	n.a.	n.a.	1q21.1q21.2 (146,503,349–147,819,438) × 3	GJA5; GJA8	Autism and focal motor epilepsy; hypertelorism; poor language skills; severe macrocephaly	Gourari et al ^[19]^
11	F /n.r.	1q21.1q21.2	1.754Mb	Paternal inherited	n.a.	1q21.1q21.2 (146074031–147828030) × 3	GJA5; GJA8	Bilateral thumb; first metacarpal hypoplasia; congenital hip; dysplasia, scoliosis, dental anomalies, short stature	Vergult et al ^[20]^ case 1
12	F/n.r.	1q21.1q21.2	1.754Mb	Paternal inherited	n.a.	1q21.1q21.2 (146074031–147828030) × 3	GJA5; GJA8	Bilateral radius dysplasia; Wassel type I and II; anomalies of the fifth finger	Vergult et al ^[20]^ case 2
13	M/TOP	1q21.1q21.28p23.3	1.796Mb1.2 Mb	n.a.	46, XY	1q21.1q21.2 (146023922–147820342) × 38p23.3 (158048–1358698) × 1	GJA5; GJA8;FBXO25; DLGAP2	G2P1; abnormal childbearing history; 25 weeks’ ultrasonic indication: short nasal bone	Our case 1
14	M/1m	1q21.1q21.2	1.242 Mb	Paternally inherited	46, XY	1q21.1q21.2 (146602934–147844778) × 3	GJA5; GJA8	G2P0A1; 23 weeks’ sonography findings: absent nasal bone, VSD, circulor of umbilical cord	Our case 2

Furthermore, we summarized the comparable cases overlapping duplicated CNVs of 1q21.1q21.2 (chr1: 146023922–147844778) in the DECIPHER database (72 cases) and the ISCA database (92 cases). The proportions of pathogenicity were as follows: pathogenic (77/164), likely pathogenic (43/164), uncertain (13/164), likely benign (1/164), and unknown (31/164). The pathogenic/likely pathogenic individuals accounted for more than 70% in all cases, which might infer high pathogenicity of this chromosomal anomaly.

Genetic disorders related to mental retardation, congenital abnormalities, autism or other clinic phenotypes could result from the alternation of gene dosage owing to chromosomal gains or losses.^[21]^ According to the DECIPHER database, a total of 11 OMIM genes exist in the region of 1q21.1q21.2, two of which are morbid genes with clinical diseases (Table 2). According to the ClinGen database, there is no available pathogenic evidence for triplosensitivity associated with the genes involved. Based upon the functions and implications, we delineated the potentially pathogenic genes which might explain the observed abnormalities and predict the possible phenotypes in future.

**Table 2:** Genes in the region of 1q21.1q21.2 based upon our cases.

Gene	OMIM	Description	Disease
GJA5	121013	gap junction protein alpha 5	Atrial standstill, digenic (GJA5/SCN5A), Atrial fibrillation, familial, 11
GJA8	600897	gap junction protein alpha 8	Cataract 1, multiple types
NBPF11	614001	NBPF member 11	N.R.
NBPF12	608607	NBPF member 12	N.R.
PRKAB2	602741	protein kinase AMP-activated non-catalytic subunit beta 2	N.R.
FMO5	603957	flavin containing dimethylaniline monoxygenase 5	N.R.
CHD1L	613039	chromodomain helicase DNA binding protein 1 like	N.R.
BCL9	602597	BCL9 transcription coactivator	N.R.
ACP6	611471	acid phosphatase 6, lysophosphatidic	N.R.
GPR89B	612806	G protein-coupled receptor 89B	N.R.
NBPF24	614001	NBPF member 11	N.R.

*GJA5* (OMIM: 121013), containing three exons, encodes a gap junction protein connexin 40 which is expressed in the right ventricular outflow tract.^[22]^ It plays critical roles in cell adhesion, intercellular communication and heart development.^[13]^*GJA5* was regarded as a candidate gene which was implicated in the etiology of cardiovascular diseases in patients carrying chromosomal 1q21.1 duplication/deletion. Duplications and mutations of *GJA5* were associated with Tetralogy of Fallot (TOF).^[23,24]^ Soemedi et al ^[25]^ observed that duplication of *GJA5* alone is associated with an 10-fold increase in the risk of TOF. In addition, mutations of *GJA5* were also detected in patients with arrhythmias.^[23]^ And dosage alterations of *GJA5* might be responsible for cardiomyopathy.^[15]^ As the flanking gene of *GJA5*, the abnormal expression of *GJA8* (OMIM: 600897) has also been correlated with congenital heart disease (CHD).^[14,15,25]^ In addition, *GJA8* was associated with eye abnormalities and schizophrenia.^[9,26]^ We speculated that the duplications of *GJA5* and *GJA8* might be responsible for the occurrence of ventricular septal defect in case 2.

*CHD1L* (OMIM: 613039), encoding a DNA helicase for chromatin-remodeling, can regulate chromatin by interacting with poly (ADP-ribose) following the process of DNA repair. High expression of *CHD1L* can be observed in different brain regions, especially in the cerebellum.^[16,19]^ Dou et al^[12]^ assumed that *CHD1L* can promote neuronal differentiation in human embryonic cells and affect nervous system development. In addition, overexpression of *CHD1L* was closely associated with patients with TOF, double outlet right ventricle, and pulmonary artery stenosis.^[27]^ Besides, *CHD1L* might also be implicated in autism spectrum disorder, attention deficit hyperactivity disorder and language dysfunction.^[17]^

*PRKAB2* (OMIM: 602741) encodes the β2-subunit of AMP-activated protein kinase (AMPK), which is a serine/threonine protein kinase activated by various cellular stimuli. It is highly expressed in cardiac muscle and plays critical roles in brain function and energy metabolism.^[5,28]^ Moreover, *PRKAB2* might have close association with schizophrenia.^[17]^ Since research on other genes in this 1q21.1 region are scarce, their potential functions and effects remain to be further investigated.

In addition, the CMA also detected a 1.2 Mb deletion in 8p23, which contained two critical genes: *FBXO25* (OMIM: 609098) and *DLGAP2* (OMIM: 605438). According to the ClinGen database, there are no available pathogenic evidence for haploinsufficiency associated with the two genes till now.

High diversity inter- and intrafamilial outcomes have been discovered among the members sharing the same 1q21.1 duplication, ranging from normal phenotypes to severe anomalies.^[12]^ The occurrence of a parent carrying 1q21.1 duplication without evident phenotypes makes it intractable to assess the clinic significance for offsprings with the same chromosomal duplication.^[7]^ In our study, case 2 inherited 1q21.1q21.2 duplication from his normally phenotypic father. The child presented low nasal bridge and ventricular septal defect, however, regular psychiatric, neurocognitive, motor skill, and neurologic status should be followed up and assessed. For the parents of case 1, since both of them presented with chromosomal abnormalities, preimplantation genetic diagnosis would be an appropriate choice if they intend to conceive again.

In this study, we delineated the clinic phenotypes and molecular cytogenetic findings of two prenatal cases carrying 1q21.1q21.2 microduplication. Our study not only enriches genotype-phenotype of 1q21.1 duplication in clinical setting, but also offers a better understanding for such chromosomal gains in prenatal diagnosis. Genetic counseling should be offered by clinicians based upon full consideration of phenotypic diversity, incomplete penetrance, and diverse phenotypic spectrum. And long term follows up involving postnatal development and clinical presentations should be assessed till adulthood, irrespective of whether the 1q12.1 duplication is *de novo* or inherited.

## Author contributions

**Conceptualization:** Yang Yu.

**Data curation:** Fagui Yue, Jing He.

**Formal analysis:** Fagui Yue, Xinyue Zhang.

**Funding acquisition:** Ruizhi Liu.

**Methodology:** Xinyue Zhang.

**Resources:** Yuting Jiang.

**Software:** Jing He, Yuting Jiang.

**Supervision:** Ruizhi Liu.

**Validation:** Ruizhi Liu.

**Writing – original draft:** Hongguo Zhang.

**Writing – review & editing:** Yang Yu.
